# Transcriptome profiling of differentially expressed genes in floral buds and flowers of male sterile and fertile lines in watermelon

**DOI:** 10.1186/s12864-015-2186-9

**Published:** 2015-11-09

**Authors:** Sun-Ju Rhee, Minseok Seo, Yoon-Jeong Jang, Seoae Cho, Gung Pyo Lee

**Affiliations:** Department of Integrative Plant Science, Chung-Ang University, Ansung, 456-756 Republic of Korea; Interdisciplinary Program in Bioinformatics, Seoul National University, Kwan-ak St. 599, Kwan-ak Gu, Seoul 151-741 Republic of Korea; CHO&KIM genomics, Main Bldg. #514, SNU Research Park, Seoul National University Mt.4-2, NakSeoungDae, Gwanakgu, Seoul 151-919 Republic of Korea

**Keywords:** Watermelon, RNA-seq, Male sterile, Male fertile

## Abstract

**Background:**

Male sterility is an important mechanism for the production of hybrid seeds in watermelon. Although fruit development has been studied extensively in watermelon, there are no reports on gene expression in floral organs. In this study, RNA-sequencing (RNA-seq) was performed in two near-isogenic watermelon lines (genic male sterile [GMS] line, DAH3615-MS and male fertile line, DAH3615) to identify the differentially expressed genes (DEGs) related to male sterility.

**Results:**

DEG analysis showed that 1259 genes were significantly associated with male sterility at a FDR *P*-value of < 0.01. Most of these genes were only expressed in the male fertile line. In addition, 11 functional clusters were identified using DAVID functional classification analysis. Of detected genes in RNA-seq analysis, 19 were successfully validated by qRT-PCR.

**Conclusions:**

In this study, we carried out a comprehensive floral transcriptome sequence comparison of a male fertile line and its near-isogenic male sterile line in watermelon. This analysis revealed essential genes responsible for stamen development, including pollen development and pollen tube elongation, and allowed their functional classification. These results provided new information on global mechanisms related to male sterility in watermelon.

**Electronic supplementary material:**

The online version of this article (doi:10.1186/s12864-015-2186-9) contains supplementary material, which is available to authorized users.

## Background

Male sterility allows the large-scale hybrid seed production, since it evades the need of emasculation. There are three reported types of male sterility based on the inheritance or origin: genic male sterility (GMS), cytoplasmic male sterility (CMS), and cytoplasmic-genic male sterility (CGMS) [1]. GMS is caused by defective nuclear genes, ordinarily inherited by a single recessive gene; CMS is maternally inherited and caused by the extranuclear genome of mitochondria or chloroplasts; and CGMS is derived by both cytoplasmic and nuclear genes. Male sterility is manifested in different ways based on the nature of plant species: (1) absence of stamens or production of abnormal stamens in bisexual plants or absence of male flowers in dioecious plants; (2) failure to develop normal anther sporogenous tissue; (3) abnormal microsporogenesis in deformed or viable pollen; (4) abnormal pollen maturation or inability to germinate on compatible stigmata; (5) defective anther dehiscence, but viable pollen formation; or (6) barriers other than incompatibility that prevent pollen from reaching the ovule [2].

Although male sterility is regarded as a gift of nature by reducing the labor to produce high-yield hybrids, the mechanism is still unclear in watermelon. To date, RNA-sequencing (RNA-seq) techniques helps to understand the global gene expression patterns and infer the candidate genes relating the male sterility mechanisms. RNA-seq analysis of male sterile floral organs have been reported in many plants such as tomato [3], cotton [4, 5], chili pepper [6], Brassica napus [7, 8], soybean [9] and so on.

Watermelon (*Citrullus lanatus* (Thunb.) Matsum. & Nakai) belongs to the family of Cucurbitaceae, which is one of the most economically important crops in the world. It has 11 chromosomes and a haploid genome of approximately 425 Mb (2*n* = 2× = 22). Watermelon genome sequence was released in 2013 [10], followed by cucumber [11] and melon [12], which makes it possible to apply next-generation sequencing (NGS). To date, some watermelon male sterile mutants, such as glabrous male-sterile (*gms*) [13–15] and male sterile dwarf (*ms-dw*) [16] *ms-1* [17], *ms-2* [18], and *ms-3* [19], have been reported. Watermelon has two flowering patterns; one is monoecious, having male and female flowers on the same plant and the other is andromonoecious, having both male flowers and hermaphrodite flowers on the same plant [20]. Male flower consists of sepal, petal, and stamen and produces pollen grains in the anther.

Anther development is a complex process that has sequential phases to produce male gametes. The anther is composed of several cell types required for pollen development including the epidermis, endothecium, and tapetum that surround the microsporocyte [21, 22]. Anther development is composed of two phases [23]. Phase 1 is defined by cell specification, tissue differentiation, and anther morphology establishment. At the early stages of anther development, archesporial cells are divided into two cell types: the primary parietal cells and the sporogenous cells. The primary parietal cells differentiate to epidermis, endothecium, middle layer, and tapetum (from outer to inner). The sporogenous cells give rise to pollen mother cell and then undergo meiosis to generate the haploid tetrads of microspores. Phase 2 is defined by pollen development, anther enlargement, tapetum/callose tissue degeneration, anther dehiscence, and pollen grain release [23, 24]. Male sterile mutants show disruption of genes related to anther and pollen development [3, 25].

Previous studies identified several genes that are related to male sterility. *SPOROCYTELESS (SPL)/NOZZLE (NZZ)* encodes a protein related to MADS box transcription factors and is required for cell differentiation and division of anther cell walls in *Arabidopsis* [26–29]. *MALE STERILITY 1* (*MS1*) [30–33] and MYB family members [34–37] are important for the tapetum and pollen development *Arabidopsis*. A basic helix-loop-helix (bHLH) transcription factor, *ABORTED MICROSPORES* (*AMS*), and *DYSFUNCTIONAL TAPETUM 1* (*DYT1*) are required for tapetum biosynthesis and microspore development in rice [38] and *Arabidopsis* [39]. A deficiency of *AtMYB103* gives rise to complete male sterility, because callose degeneration, tapetal development, and exine formation during anther development are interrupted [36]. These key genes related to anther development from archesporial tissue development to pollen grain release are responsible for male sterility.

Transcriptome profiling showed similar patterns of functional categories, such as cell wall modification and metabolism, cell cycle regulation, hormonal regulation, energy metabolism, pollen development and cytoskeleton dynamics, between male sterile and fertile lines [4, 40, 41]. In Cucurbitaceae, transcriptome profiling data of floral development are limited to floral sex determination [42, 43]. These studies suggested that ethylene is the most important hormone for regulating female sex determination in cucumber and that auxin, abscisic acid, and brassinosteroids may have an effect on sex determination through their interaction with ethylene. While many transcriptome analyses have been performed in various plant species, only a few have been conducted on fruit development, fruit color, and incompatible interaction with pathogen in watermelon [44–50].

In this study, we focused on the detecting differentially expressed genes (DEGs) related to male sterility. In order to this, RNA-seq was performed with floral buds and mature flowers from two different watermelon lines [genic male sterile (GMS) line, DAH3615-MS, and male fertile line, DAH3615], because two different stages of organs would be reduced to make a false decision for understanding the mechanisms. In addition, significantly detected DEGs across the RNA-seq analysis were technically validated by real-time reverse transcription polymerase chain reaction (qRT-PCR).

## Results

### Pre-processed RNA-seq data

Raw RNA-seq data were pre-processed using Trimmomatic [51] for generating clean reads. As shown in Table 1, an average of 12,645,328 (94.09 %) surviving reads was retrieved from each data set. All clean reads were mapped on the reference genome of watermelon (cv. 97103) [10]. The overall mapping rate ranged from 58.3 to 62.2 % and the concordant alignment rate from 51 to 54.7 %. A total of 23,440 gene abundances were measured using HTSeq [52] and 19,732 genes remained after filtering out non-expressed genes across all samples.

**Table 1:** Pre-processed RNA-sequencing data using Trimmomatic

Sample	# of surviving reads	Surviving reads	Overall mapping rate	Alignment rate
Mf_bud	11,834,220	93.55 %	62.2 %	54.7 %
Mf_flower	13,845,808	93.90 %	60.3 %	52.9 %
Ms_bud	12,411,872	94.64 %	61.1 %	53.7 %
Ms_flower	12,489,412	94.25 %	58.3 %	51.0 %

**Fig. 1:**
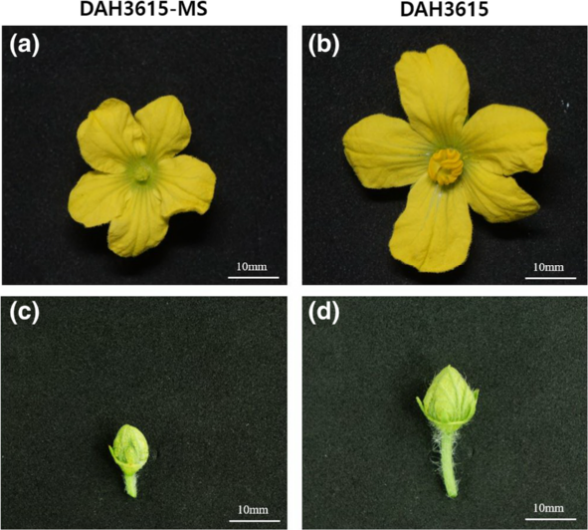
Morphological differences in floral organs of a male sterile line (DAH3615-MS) and a male fertile line (DAH3615). The flower (a) and floral bud (**c**) of the male sterile line, DAH3615-MS, are smaller than those (**b**, **d**) of the male fertile line, DAH3615. The male sterile flower (**a**) shows distinctive defective stamen and absence of pollen grains. The images were taken at 63 d after sowing

Each sample represents a floral organ in a breeding line; *Mf* male fertile line (DAH3615), *Ms* male sterile line (DAH3615-MS), *bud* floral bud, and *flower* mature flower, as shown in Fig. 1. Including all statistics was calculated based on the paired-end reads

### Identification of DEGs

**Fig. 2:**
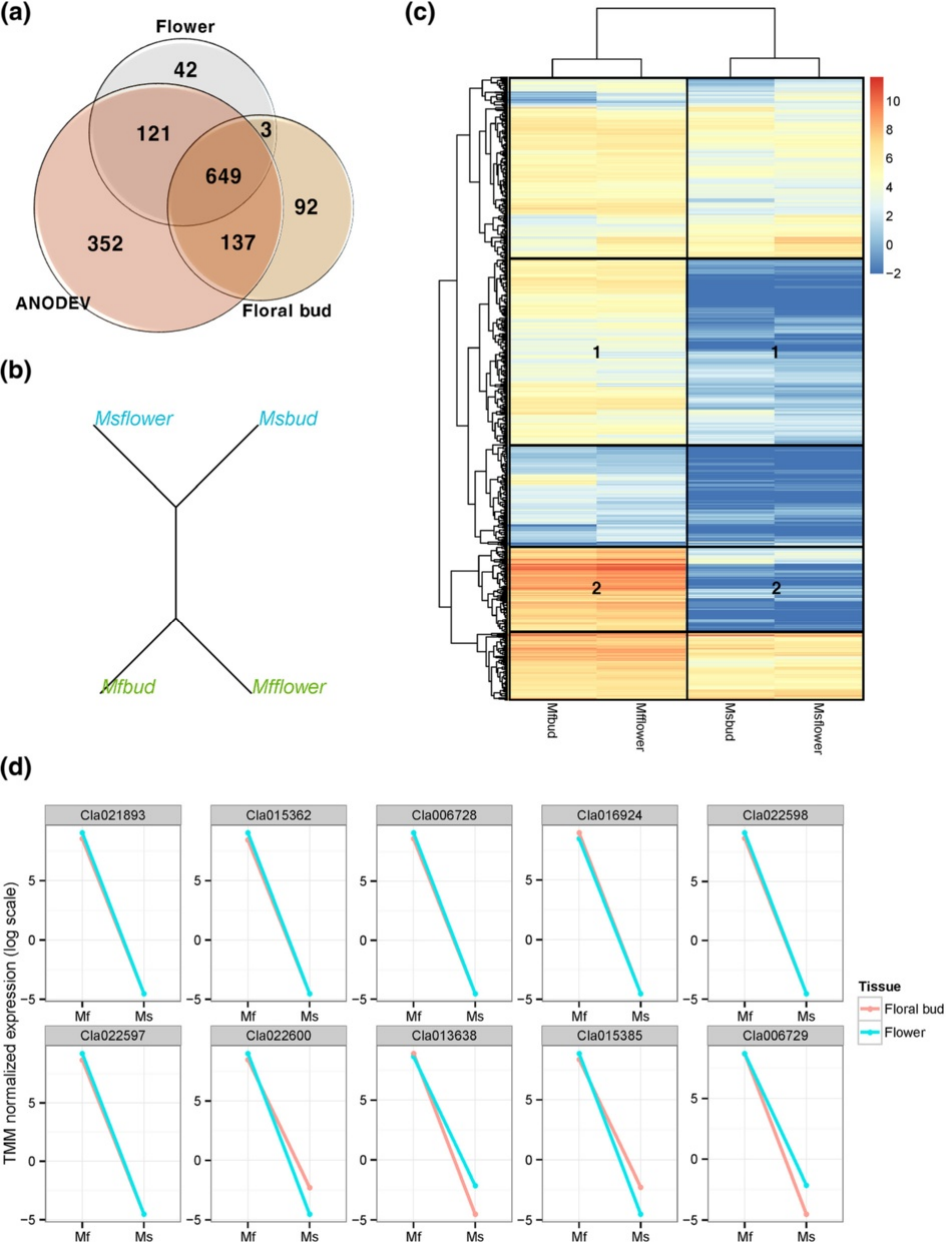
Identification of differentially expressed genes (DEGs) in a male sterile line (DAH3615-MS) and a male fertile line (DAH3615). (**a**) Venn-diagram of significantly different DEGs detected in the male fertile line and the male sterile line using two types of statistical tests such as Fisher’s exact test and 2-way ANODEV [False discovery rate (FDR) adjusted *P*-value < 0.01]. In the Fisher’s exact test, two tissues’ samples, bud and floral bud, were respectively employed. (**b**) Hierarchical clustering analysis tree with *k* = 2. Blue and green lines represent DAH3615-MS and DAH3615, respectively. (**c**) Heatmap with hierarchical clustering using logged TMM-normalized gene expressions of the 1259 DEGs (FDR adjusted *P*-value < 0.01) from the analysis of deviance (ANODEV). Regions 1 and 2 show highly significant differences between DAH3615-MS and DAH3615. (**d**) Line plots show the gene expression of the top 10 genes from ANODEV. Red and blue lines represent floral bud and flower, respectively. The y-axis represents logged TMM-normalized gene expressions, which were calculated in the *edgeR*

We compared mRNA expression in floral buds and flowers of DAH3615 and DAH3615-MS samples (Fig. 1) using two different statistical tests [analysis of deviance (ANODEV) and Fisher’s exact test], in order to detect sterility-related genes (Fig. 2a and Additional file 1). A total of 649 DEGs were identified from both tests in both organs (floral bud and flower). The highest total number of DEGs [1259; false discovery rate (FDR) adjusted *P*-value <0.01] was revealed by ANODEV. Out of 1259 DEGs, 352 were exclusively detected by ANODEV. Fisher’s exact test revealed 42 and 92 DEGs in the floral bud and flower, respectively, with a lower FDR adjusted *P*-value than that in ANODEV. Overall, ANODEV showed a higher statistical power than Fisher’s exact test, and we decided to focus on the DEGs detected from the former test. Hierarchical clustering analysis was performed with *k* = 2 and results are shown in Fig. 2b. DAH3615 and DAH3615-MS were clearly distinguishable, since strong differences were observed between the lines and not between the organs. The relationship of all the significant DEGs was visualized in a heatmap as shown in Fig. 2c. Two regions (Region 1 and 2) were highly different between DAH3615 and DAH3615-MS. In these regions, DEGs showed patterns that were only identified in DAH3615, regardless the organ. In order to further investigate these results, line plots of the top 10 genes were drawn (Fig. 2d) and confirmed that these genes had similar patterns that were only expressed in DAH3615.

### Male sterility related DEGs in relation to biasness of gene expression and chromosomal location

**Fig. 3:**
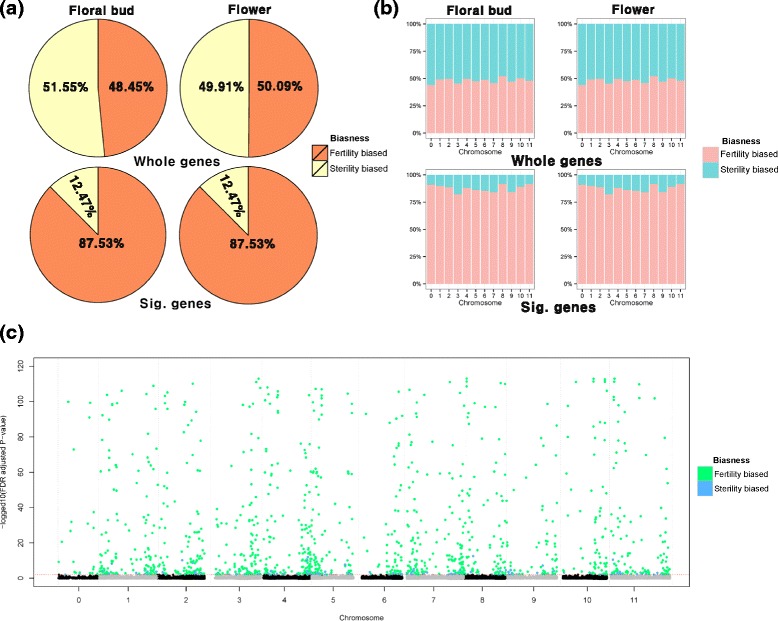
Characterization of significantly detected differentially expressed genes (DEGs) using chromosomal location and biasness. Fertility biased: DAH3615-biased expression; Sterility biased: DAH3615-MS-biased expression. (**a**) Pie charts show the biasness of significantly detected DEGs. The upper two proportional pie charts were created using the whole-annotated genes and the lower two pie charts were created using 1,259 DEGs from the analysis of deviance (ANODEV). (**b**) Βiasness of significantly detected genes. (**c**) Manhattan plot represents the chromosomal location of significantly detected genes. The y-axis represents *log*_10_ false discovery rate (FDR) adjusted *P*-value. The red dotted line is a cut-off line (FDR adjusted *P*-value < 0.01)

DEG analysis showed that most of the detected genes were exclusively expressed in DAH3615; hence, the trend of gene expression was expected to be skewed towards male fertility. Detected DEGs were characterized using biasness information, calculated as the differences between DAH3615 and DAH3615-MS in floral buds and flowers using the log expression ratios (trimmed mean of M values). As shown in Fig. 3a, approximately 50 % of the genes in DAH3615 and DAH3615-MS were biased in both organs, when considering gene expressions of the whole genes. However, analysis of the significantly detected DEGs showed that 87.53 % of the genes were DAH3615-biased and only 12.47 % of the genes were DAH3615-MS-biased in both organs. Moreover, the DAH3615-biased genes in the floral bud were also biased in the flower. These results indicate that the gene expression pattern of the detected genes was maintained from the floral bud to the flower.

We also investigated the chromosomal location of the detected sterility-related genes using the watermelon reference genome that is composed of 12 chromosomes including chr 0 (undetermined chromosome). The biasness of the significantly detected genes was investigated as shown in Fig. 3b. We observed that the sterility-related genes were not clustered in specific chromosomes, but they were uniformly distributed along the genome. To further investigate these results, the significantly detected genes were visualized in a Manhattan plot as shown in Fig. 3c. The Manhattan plot is usually used in genome-wide association studies (GWAS) to investigate the chromosomal location of single nucleotide polymorphisms (SNPs). It can be applied in RNA-seq analysis because the location of each gene is also known, based on the reference genome. Therefore, the representative location value of each gene can be determined by a Manhattan plot from the annotated mRNA central position on the reference genome. The plot showed a strong signal originated by the DAH3615-biased genes, and that the telomere of each chromosome was enriched to sterility related genes.

### Gene-set enrichment analysis for functional annotation of significantly detected sterility-related genes

In order to investigate the biological function of the 1259 detected genes (1105 and 154 genes in DAH3615 and DAH3615-MS biased expression, respectively), gene-set enrichment analysis was performed by DAVID tool using the default stringency level (medium), which is considered [53]. Since only a few studies were performed in watermelon, we used *Arabidopsis* gene annotation as a background database, which is relatively well composed. As a result, DAVID analysis with 1105 DAH3615 biased genes showed that many significantly enriched biological terms including gene ontology (GO) and INTERPRO terms as shown in Table 2. On the other hand, no significant terms were detected in DAVID analysis with the detected 154 DAH3615-MS biased genes. In the Table 2, 11 clusters with their enrichment scores (ES) were reported from the functional annotation clustering analysis. Cluster 1 included copper binding sites, cupredoxin, and multicopper oxidases (type 1, 2, and 3). Cluster 2 and 4 included several lipid-related terms, while cluster 3 several kinesin-related terms and activity-related terms in microtubule. Cluster 5 included signal-related biological terms, while cluster 6 included the highest number of biological terms such as kinase-related terms, ATP-related terms, and nucleotide-binding-related terms. Cluster 7 included diverse development-related terms, while cluster 8 included membrane- and topology-related terms. Cluster 9 and 10 included several binding related terms. Finally, Cluster 11 included DNA- and RNA-related terms. Functional classification analysis using significantly detected DEGs successfully revealed the associated function of the fertility-related genes.

**Table 2:** DAVID functional classification analysis

Cluster	Terms	ES
Cluster 1	Cupredoxin, copper ion binding, Multicopper oxidase, type 3, Multicopper oxidase, type 2, Multicopper oxidase, type 1	2.25
Cluster 2	Proteoglycan, lipid moiety-binding region: GPI-anchor amidated serine, lipoprotein, propeptide: Removed in mature form, GPI-anchor, cell membrane, glycoprotein	1.35
Cluster 3	Kinesin, motor region, conserved site, KISc, Kinesin, motor region, microtubule associated complex, microtubule motor activity, motor protein, microtubule-based movement, microtubule, motor activity, microtubule-based process, microtubule, microtubule cytoskeleton, cytoskeletal part, cytoskeleton, intracellular non-membrane-bounded organelle, non-membrane-bounded organelle	1.13
Cluster 4	Secondary metabolic process, cellular amino acid derivative metabolic process, phenylpropanoid metabolic process	0.95
Cluster 5	Signal peptide, signal, glycoprotein, lycosylation site:N-linked (GlcNAc…), extracellular region, Secreted	0.94
Cluster 6	protein serine/threonine kinase activity, Serine/threonine protein kinase, active site, serine/threonine-protein kinase, protein amino acid phosphorylation, phosphate metabolic process, phosphorus metabolic process, protein kinase activity, receptor	0.83
	kinase, phosphorylation, Protein kinase, core, protein tyrosine kinase activity, Protein kinase, ATP binding site, nucleotide-binding, atp-binding, ATP binding, adenyl ribonucleotide binding, ribonucleotide binding, purine ribonucleotide binding, Serine/threonine protein kinase-related, adenyl nucleotide binding, purine nucleoside binding, nucleoside binding, purine nucleotide binding, nucleotide binding	
Cluster 7	Developmental growth involved in morphogenesis, unidimensional cell growth, developmental growth, cell morphogenesis, cell growth, regulation of cell size, cellular component morphogenesis, regulation of cellular component size, growth	0.7
Cluster 8	Intrinsic to membrane, topological domain: Cytoplasmic, topological domain: Extracellular, membrane, transmembrane region, transmembrane, integral to membrane	0.36
Cluster 9	Cation binding, ion binding, metal ion binding, transition metal ion binding	0.33
Cluster 10	Transition metal ion binding, oxidation reduction, oxidoreductase, metal-binding	0.09
Cluster 11	Intracellular signaling cascade, transcription regulation, nucleus, Transcription, response to endogenous stimulus, transcription, regulation of transcription, DNA-dependent, regulation of RNA metabolic process, response to organic substance, response to hormone stimulus, dna-binding, transcription factor activity, transcription regulator activity, regulation of transcription, DNA binding	0.08

DAVID functional annotation analysis was performed using 1,105 DAH3615-biased genes derived from the RNA analysis. Twelve clusters were identified including their enrichment score (ES)

### Technical validation of detected fertility-related genes using qRT-PCR

**Fig. 4:**
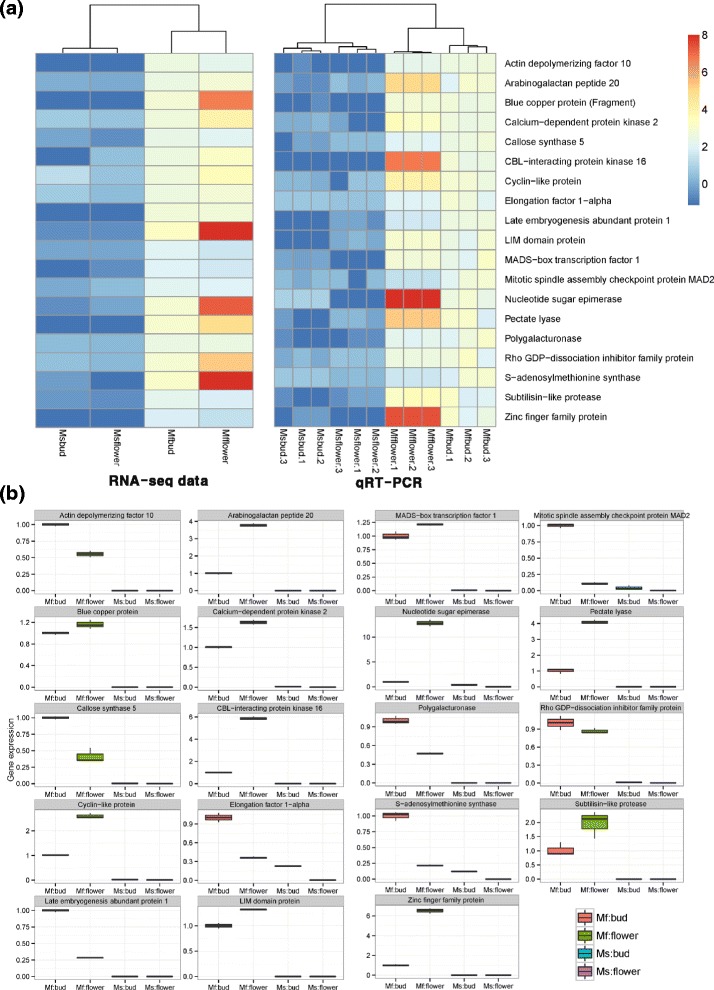
Technical validation of 19 randomly selected differentially expressed genes (DEGs) detected by RNA-sequencing (RNA-seq) using qRT-PCR. (**a**) Relative heatmaps of RNA-seq and qRT-PCR. Gene expression from the two platforms was normalized by the quantile normalization method. (**b**) Box-plots of 19 DEGs detected by qRT-PCR. The y-axis represents the gene expression level, which is the **−**2^∆Δ*Ct*^ value of qRT-PCR compared to the control gene

As the RNA-seq experiment was performed without biological replicates, we have conducted verification experiment with several biological replicated samples in order to confirm the determination of DEGs. Although RNA-seq is more accurate than previous known chip-based transcriptome measuring platforms, such as microarray, biological replication was still important to accurately estimate the expression mean and variance in each group. Many RNA-seq based transcriptome studies have been carried out without biological replications, because RNA-seq is often used for pre-screening, in order to reduce the candidate genes for qRT-PCR. The qRT-PCR methods has been traditionally used for measuring gene expression level, but it can measure gene expression relatively more accurately than high-throughput platforms such as RNA-seq and microarray. In our study, the first RNA-seq analysis was performed, in order to select candidate genes for qRT-PCR. A total of 21 genes were randomly selected from the significantly detected DEGs in the RNA-seq analysis (FDR adjusted *P*-value < 0.01). We performed qRT-PCR using these 21 genes with 3 biological replicates from each group, namely male fertile floral bud, male fertile flower, male sterile floral bud, and male sterile flower (*n* = 12). Of 21 genes, 19 were successfully measured by qRT-PCR, while 2 genes (calmodulin- and fasciclin-like arabinogalactan protein 17) failed to reach the threshold (Additional file 2). Most of tested genes showed apparent expression in the floral buds and flowers of DAH3615, but no or slight expression in those of DAH3615-MS. Of these genes, S-adenosylmethionine synthase and elongation factor 1-alpha were reduced by approximately 10-fold and 5-fold in the floral buds of DAH3615-MS and those of DAH3615, respectively, but not detectable in DAH3615-MS flowers. DEGs of DAH3615 and DAH3615-MS were consistent with RNA-seq data. In order to visually compare RNA-seq and qRT-PCR results, the quantile normalization method was used for adjusting different scales of gene expression. Based on the normalized gene expression data, relative heatmaps were generated as shown in Fig. 4a. In both RNA-seq and qRT-PCR, clear separation was depended on the breeding line, and the patterns of hierarchical clustering had high similarity. In addition, differences between the biological replications were very small in qRT-PCR as shown in Fig. 4a and b. Student’s *t*-test was used to separate the differences between DAH3615 and DAH3615-MS lines, and the results are presented in Table 3. Based on these results, we confirmed that the 19 genes were fertility-related genes and that RNA-seq was highly consistent. In addition, the male fertility biased gene expression pattern was also technically validated by qRT-PCR.

**Table 3:** qRT-PCR test results of 19 genes detected by RNA-sequencing

Gene description	Uniport_ID	Floral Bud	Flower
Actin depolymerizing factor 10	B9I489_POPTR	1.35E-04***	2.44E–03**
Arabinogalactan peptide 20	AGP20_ARATH	8.00E–04***	2.10E–04***
Blue copper protein (Fragment)	O82576_MAIZE	1.87E–04***	1.56E–03**
Calcium–dependent protein kinase 2	Q3YAT0_PETIN	1.10E–04***	4.03E–04***
Callose synthase 5	C8C9X3_ARATH	8.66E–05***	2.26E–02*
CBL–interacting protein kinase 16	C4P7W5_VITVI	2.92E–04***	1.92E–04***
Cyclin–like protein	Q6ZIX9_ORYSJ	2.59E–05***	6.36E–04***
Elongation factor 1–alpha	B9SPV9_RICCO	2.75E–03**	7.44E–04***
Late embryogenesis abundant protein 1	LEA1_CICAR	1.41E–04***	2.35E–04***
LIM domain protein	Q306K1_BRANA	8.75E–04***	5.13E–06***
MADS–box transcription factor 1	D1MFS6_HEVBR	1.99E–03**	6.63E–05***
Mitotic spindle assembly checkpoint protein MAD2	D2V3A0_NAEGR	5.34E–06***	8.07E–03**
Nucleotide sugar epimerase	B1WNM2_CYAA5	1.02E–02*	8.12E–04***
Pectate lyase	B6TSP4_MAIZE	7.71E–03**	2.84E–04***
Polygalacturonase	E3VSV7_CUCPE	1.42E–03**	1.96E–04***
Rho GDP–dissociation inhibitor family protein	D7L724_ARALL	4.51E–03**	9.35E–04***
S–adenosylmethionine synthase	B9S0G1_RICCO	2.14E–03**	6.32E–04***
Subtilisin–like protease	Q6WNU4_SOYBN	1.86E–02*	1.93E–02*
Zinc finger family protein	D7MD22_ARALL	3.19E–03**	5.41E–04***

Student’s *t*–test was used for separating the differences between a male sterile line (DAH3615–MS) and a male fertile line (DAH3615) in floral buds and flowers. *P < 0.05, **P < 0.01, ***P < 0.001

## Discussion

GMS is caused by abnormal gene expression in several developmental stages of stamen development, male gametophyte development, pollen formation, pollen nutrient support, and pollen tube elongation. Previous studies used transcriptional profiling or proteomic analysis to detect DEGs from male sterile and male fertile lines [5, 6, 8, 54]. In this study, we performed RNA-seq and compared transcripts of floral buds and flowers between a male sterile line, DAH3615-MS, and its near-isogenic fertile line, DAH3615, in watermelon. Overall, 1259 genes were detected as DEG between DAH3615 and DAH3615-MS. Of detected genes, 1105 genes were identified as only expressed in DAH3615 and they were annotated into 11 clusters’ biological terms by DAVID analysis (Table 2). These clusters included copper-ion binding sites (cluster 1), lipid metabolism terms (cluster 2), cytoskeleton terms (cluster 3), cellular metabolism terms (cluster 4), signaling terms (cluster 5), kinase-related terms (cluster 6), developmental growth terms (cluster 7), membrane location terms (cluster 8), ion-binding terms (cluster 9), oxidation reduction terms (cluster 10), and DNA- and RNA-related terms (cluster 11). For validating RNA-seq data obtained by qRT-PCR, we selected 21 genes from each cluster. Of those, 19 showed consistent results by both platforms (Fig. 3). In this study, DEGs were involved in stamen development, and pollen development, formation, and germination.

A previous study showed that significant genes related to programed cell death (PCD) are expressed spatially and temporally for tapetum degeneration and anther dehiscence [55]. In this study, DEGs for PCD were subtilisin-like protease, disease resistance-responsive (Dirigent-like) family protein, and cysteine proteinase found in cluster 7. Subtilisin-like proteinase is expressed at the tetrad stage in lily[56] and up-regulated in the floral bud of wild-type cabbage [57], while 5B-protein, an anther-specific protease, is associated with the degradation of regulatory proteins in tomato anthers [58]. Subtilisin-like proteinase and dirigent-like protein are involved in lignin biosynthesis and participate in anther dehiscence [59].

Genes related to cell wall loosening were prominently expressed in DAH3615 and included in cluster 7 such as polygalacturonase (PG), expansins, pectate lyase, cellulase (glycosyl hydrolase family 5), endoglucanase, and glucan endo-1 3-beta-glucosidase 5. PG is involved in anther dehiscence [60, 61], pollen mother cell wall degradation [62], pollen intine and exine formation [63], and pollen tube growth [64], while pectate lyase in pollen wall development [65, 66] and pollen tube growth [67, 68]. In this study, male-sterile flowers did not produce any pollen, since many genes related to pollen formation, development, and pollen tube growth were distinguishably expressed in the floral buds and flowers of DAH3615, but not in those of DAH3615-MS.

Callose, beta-1, 3-glucan, is a polysaccharide that separates developing pollen grains, preventing their walls from fusion. The callose wall is temporarily synthesized by callose synthase 5 (Cal5) that requires microspore callose deposition to prevent cell fusion [69, 70] and for this reason Cal5 knockout mutant shows reduced fertility [71]. Callose wall should be timely broken down to release the microspores into the locules. Thus, the mistiming of callose degradation leads to male-sterility. Beta-1, 3-glucanase (callase), a PCD-related gene expressed in the reproductive organs, is secreted from tapetum cells and causes callose degradation [24]. In this study, arabinogalactan proteins (AGPs), such as GPI-anchored proteins, COBRA-like proteins encoding GPI-anchored proteins, AG peptide 20 and 23, and fasciclin-like AGP 17, were annotated in the DEG data. AGPs participate in cell expansion, division, seed germination, and pollen tube growth and guidance [72].

Calcium-gradient mediated pollen tube growth is one of the best-characterized metabolism systems [73]. In this study, many genes of calcium-gradient mediated pollen tube growth were expressed in DAH3615 such as calcium-dependent protein kinase 25, Rho GDP-dissociation inhibitor family protein, CALCINEURIN B-LIKE (CBL)-interacting protein kinase 16, calmodulin, and calmodulin-like (CaM) proteins. Calcium-binding proteins act as calcium sensors and relay calcium signals. Major calcium sensors have been reported such as calcium-dependent protein kinase, CaM, CBL proteins, and CML protein [73]. In *Arabidopsis*, CIPK19 is expressed specifically in pollen grains and controls pollen polarity [74]. Rho GDP-dissociation inhibitor protein isolated from *Nicotiana tabacum* (Nt-RhoGDI2) is specifically expressed in elongating tobacco pollen tubes and inhibits the formation of transversal actin bands [75].

Defective cytoskeleton dynamics is also one of the factors causing male sterility [76]. In this study, genes related to cytoskeleton were included in cluster 3. Male-sterile lines of wheat and Chinese cabbage have much less actin in the anther than the fertile wild-type plants. Therefore, it has been suggested that low actin levels in the anther are associated with male sterility [77]. LIM domain-containing proteins, which are classified in the novel family of actin bundling proteins, act as transcriptional activators of lignin biosynthesis. Several LIM domain-containing proteins are specifically expressed in the pollen [78]. A previous study showed that the PLIM2c promoter initiated its expression in the pollen during stamen filament elongation and the PLIM2c protein was expressed in the developing pollen grains of *Arabidopsis*. In addition, the PLIM2c-knockout mutant had sterile pollen [79]. Actin depolymerizing factor (ADF), an actin binding protein, affects cytoskeleton architecture dynamics that play a role in the regulation of F-actin filament assembly, which is involved in the polarized tip growth by regulating the actin cytoskeleton in *Arabidopsis* [80, 81].

The final developmental stage of pollen maturation includes a programmed desiccation process for enhancing pollen-geminating efficiency. Therefore, pollen grains have desiccation tolerance during pollen maturation [82]. Dehydrins or late embryogenesis abundant (LEA) proteins protect pollen from desiccation [83]. In this study, the related genes (stress-induced genes) were included in cluster 9.

MADS box transcription factor 1 and zinc-finger family proteins were included in cluster 11. MADS-box transcription factor controls the specification of stamen primordia [84], while several zinc-finger family proteins play important roles in flower development. The latter proteins are especially activated during anther development in petunia [85].

Development of the male reproductive organ includes sporophytic cell division through mitosis and male gametogenesis through meiosis. Therefore, mitotic and meiotic-specific gene destruction during cell division process can lead to male sterility [86]. In this study, cyclin-like protein, kinesin-like protein, and mitotic spindle assembly checkpoint protein MAD2 were differentially expressed in DAH3615 than in DAH3615-MS.

Interestingly three DEGs in our result have scant information in male sterility related previous reports.

Elongation factor-1 alpha (EF-1α) is a well-known housekeeping gene, which is expressed in whole organs including reproductive organs. In vivo assay which visualization of EF-1α using EF-1α-Gus expressed transgenic tobacco plant showed Gus expression in meristems of shoot, root and floral organs [87]. However, our RNA-seq results and qRT-PCR validation in this study were deduced to absence in male sterile flower buds and flowers. It has a previously report that proteome analysis using *Zea mays* ms8 mutant anther have shown that EF-1α as a differentially expressed protein [88]. EF-1α is a multi-functional protein which play a role such as elongation of translation, cytoskeleton regulation, and signal transduction. Additionally, it is participated in interaction of calmodulin [87], substrate of calcium dependent protein kinases [89] and Rho associated kinase [90], and putative regulator of DNA replication/ repair protein [91]. Most of these EF-1α functions are deduced in our DEG results. Therefore, EF-1α is crucially required for the successful development of male reproductive organ of watermelon.

Blue copper protein was the most significant expressed gene that included in cluster 1, which was the most enriched cluster. Plantacyanins, a sub-subfamily of phytocyanins that belongs to type 1 ‘blue copper protein’, are secreted from pistil and guide pollen and pollen tube to interact with the pistil. Although there is limited information whether blue copper protein is associated with male sterility, transcriptome analysis showed that blue copper protein is down regulated in CMS cotton line [4]. So, blue copper protein may be involved in determination of the male sterility in watermelon, but it would be required for progress studies.

In cucumber, cDNA subtractive hybridization using gynoecious and hermaphrodite floral buds and flowers has shown that nucleotide sugar epimerase may be involve in stamen development [92]. Nucleotide sugar epimerase showed low expression in DAH3615-MS. Even though the gene has little information related to floral organ development or male sterility, the result of our RNA-seq data provides possibility of the gene would be a putative novel gene which implicates for inducing male sterility by affect cell expansion, especially of stamen primordia.

## Conclusions

In conclusion, we successfully detected 1259 DEGs (FDR adjusted *P*-value < 0.01) by comparing floral buds and flowers of a male-sterile line (DAH3615-MS) and a male-fertile line (DAH3615) using two-way ANODEV. To our knowledge, this is the first report on transcriptional profiling in watermelon for identifying DEGs related to male sterility and investigating expression patterns in male-sterile and fertile reproductive organs. We also anticipated some functions, such as stamen development, pollen formation, and pollen tube elongation, of essential genes in the fertile male flower that referred in previous reports. Our results provide new information on global mechanisms related to male sterility in watermelon.

## Methods

### Plant materials

Floral buds and flowers were harvested from progenies of an 1:1 segregating watermelon population between a GMS line (DAH3615-MS, *msms*) derived from the *ms-1* Chinese male sterile line [17] and its fertile near-isogenic line (DAH3615, *Msms*), which was selected from a backcross program. The plants were grown in a greenhouse during the spring of 2014. Floral buds of DAH3615-MS and DAH3615 were collected from the 22nd–23rd node (3–4 mm and 7–8 mm in length, respectively) 1 d before flowering, while mature flowers were collected the first day of flowering from the 16–17th node. The samples were directly frozen in liquid nitrogen and then stored at −80 °C until RNA extraction.

### RNA-seq experiment

To generate RNA-seq reads, we followed Illumina sequencing protocol. Total RNA was isolated from floral buds and flowers of DAH3615-MS and DAH3615 using Tri-Reagent® (MRC, OH, USA) added 1 % mercaptoethanol according to the manufacturer’s manual. Quantification was performed using Nanodrop spectrophotometer (Thermo Scientific, USA), while quality assessment was performed with RNA 6000 Nano assay kit (Agilent, USA) using Bioanalyser 2100 (Agilent, USA). NGS sequencing libraries were generated from 1 μg of total RNA using Truseq RNA Sample Prep Kit (Illumina, USA) according to the manufacturer’s protocol. The resulting libraries were then paired-end sequenced (2x101bp) with the Illumina HiSeq™ 2000 system. Finally, FastQC V0.10.1 with ASCII Q-score offset 33 was used to check sequencing quality. More detailed QC results can be shown in Additional file 3.

### Reference genome and RNA-seq data pre-processing

We used watermelon reference genome (cv. 97103) version 1 from the Curcurbit Genomics Database. Prior to aligning, we employed Trimmomatic [51] to generate clean reads, removing Illumina adapter sequences. Clean reads were mapped on watermelon genome using Bowtie2 [93] included in Tophat2 [94]. Finally, we used HTSeq [52] along with watermelon gene annotation file from the Curcurbit Genomics Database for measuring mRNA expression. In this study, we focused only on the quantification of known mRNA sequences. Therefore, unknown proteins or isoforms were not considered in this study.

### Statistical methods for detecting male sterility related genes

Two types of statistical tests were employed for detecting DEGs, Fisher’s exact test and ANODEV. First, the Fisher’s exact test is widely used for RNA-seq analysis without replications to compare gene expressions between two groups. Employing this statistical test is easiest way for detecting DEGs in each organs, under our study design. This experiment had a 2×2 factorial arrangement, since the generated RNA-seq data had two factors with two levels each, breeding lines (male sterile line: DAH3615-MS and male fertile line: DAH3615) and organ information (floral bud and mature flower). In this situation, 2-way ANODEV model can be employed for detecting DEGs considering experimental design as follows (1):

1 $$ Expressio{n}_{{}_{ij}} = \mu + breeding\ lin{e}_i + orga{n}_j $$

where *i* = {“male fertile line (DAH3615)” and “male sterile line (DAH3615_MS)”}

and *j* = {“floral bud”, “flower”}

From the model, statistical test was performed about group effect (comparison between male sterile DAH3615-MS and male fertile DAH3615) for all genes, respectively, when organ effect was considered. Both statistical tests were performed in edgeR for detecting DEGs [95], which results were attached in Additional file 1. A gene was considered significant at FDR adjusted *P*-value of < 0.01.

### qRT-PCR experiment for technically validating detected DEGs from RNA-seq analysis

In order to technically validate the results of RNA-seq, we performed qRT-PCR Eco™ Real-Time PCR system (Illumina, CA, USA). The experiment was performed using 3 biological replicates from each group, namely male fertile floral bud, male fertile flower, male sterile floral bud, and male sterile flower (*n* = 12). Of the detected DEGs from RNA-seq analysis, 21 genes were selected using the results from previous studies on male sterility-related genes. Representative samples of two developmental stages, floral buds and flowers, from each of DAH3615-MS and DAH3615 were used to validate RNA-seq results by qRT-PCR. PCR reaction mixture was consisted of 1 μl cDNA, 10 μl pre-mix, 1 μl evergreen fluorescence dye (SolGent, Korea), and 500 nM of each primer (except for 18S reran that 250nM of each primer were used). Cycling conditions were as follows: 95 °C for 12 min, 40 cycles at 95 °C for 10 s, and 60 °C for 30 s. Watermelon 18S reran was used as an internal control for normalizing mRNA and 20 candidate genes were selected and performed by qRT-PCR for evaluating DEGs. Primers were designed by Primer3 tool [96] using gene sequences from the Curcurbit Genomics Database and presented in (Additional file 4). The relative expression levels of selected genes were calculated using the 2^−ΔΔCT^ transformation method (Additional file 2). The expression level of each transcript from the floral buds of DAH3615-MS was used as a calibrator. All reactions were performed in triplicate. The statistical analysis for qRT-PCR, which can be shown in Additional file 4.

### Availability of data and materials

Our generated RNA-seq raw data supporting the results of this article is available in the Gene Expression Omnibus (GEO) repository. The accession number is GSE69073 (http://www.ncbi.nlm.nih.gov/geo/query/acc.cgi?acc= GSE69073).

## Additional files

Additional file 1:
**RNA-seq analysis results.** The spreadsheet file contains statistical analysis results including FDR adjusted *P*-values of the 2-way ANODEV and Fisher’s exact test as well as gene annotation. (XLSX 1795 kb) Additional file 2:
**Gene expression in the qRT-PCR.** Normalized gene expression is included in the file. (XLSX 11 kb) Additional file 3:
**Quality control result for RNA-seq experiment.** The file contains QC result of the RNA-seq experiment. (XLSX 8 kb) Additional file 4:
**Detailed information for the qRT-PCR experiment.** Statistical analysis results and primer information, which is included in this file. (DOCX 21 kb)

## Abbreviations

ADF: Actin depolymerizing factor
AGP: Arabinogalactan proteins
ANODEV: Analysis of deviance
CaM: Calmodulin
CBL: CALCINEURIN B-LIKE
CGMS: Cytoplasmic-genic male sterility
CMS: Cytoplasmic male sterility
DEGs: Differentially expressed genes
EF-1α: Elongation factor-1 alpha
ES: Enrichment scores
FDR: False discovery rate
GMS: Genic male sterility
GO: Gene ontology
GWAS: Genome-wide association studies
HTSeq: High throughput sequencing
LEA: Late embryogenesis abundant
NGS: Next-generation sequencing
PCD: Programmed cell death
PG: Polygalacturonase
qRT-PCR: Quantitative reverse transcription polymerase chain reaction
RhoGDI: Rho GDP-dissociation inhibitor protein
RNS-seq: RNA-sequencing
SNPs: Single nucleotide polymorphisms
