# Supplementary material for: Transcriptome profiling of differentially expressed genes in floral buds and flowers of male sterile and fertile lines in watermelon
Source: BMC Genomics. 2015 Nov 9;16:914. doi: 10.1186/s12864-015-2186-9 (PMC4640349; doi:10.1186/s12864-015-2186-9)
Supplement: Additional file 4: — Detailed information for the qRT-PCR experiment. Statistical analysis results and primer information, which is included in this file. (DOCX 21 kb) [file 12864_2015_2186_MOESM4_ESM.docx]

Table S1. Used primers for qRT-PCR experiments

| Gene name | Uniprot_ID | 5′ → 3′ | Sequence | Product size (bp) |
| --- | --- | --- | --- | --- |
| Actin depolymerizing factor 10 | B9I489_POPTR | Forward | CTCCCTGCAGATGAGTGTCG | 113 |
|  |  | Reverse | CCTTGGAGCTTGCATACACC |  |
| Arabinogalactan peptide 20 | AGP20_ARATH | Forward | GCTGTTGCTTTGCTTTTGGC | 136 |
|  |  | Reverse | GCACCAAGGCCAACAACATC |  |
| Blue copper protein | O82576_MAIZE | Forward | GGCGTCGTCTCCAAACACTA | 130 |
|  |  | Reverse | CACTGCAGTGGAGGAGGAAG |  |
| Calcium-dependent protein kinase 2 | Q3YAT0_PETIN | Forward | CCGGGGTGAATTTGGGATCA | 128 |
|  |  | Reverse | CGACCTCTCTACGCACATCC |  |
| Callose synthase 5 | C8C9X3_ARATH | Forward | CGATGGTCATTGCTGCATGG | 109 |
|  |  | Reverse | AGAAGACGAAGGACTGCAGC |  |
| Calmodulin | B6SMJ0_MAIZE | Forward | GCGACGGTAAGATCTCTGCA | 100 |
|  |  | Reverse | GTGTCGATCTCGGCCATCAT |  |
| CBL-interacting protein kinase 16 | C4P7W5_VITVI | Forward | GAGGCCACAGTCTCGTTTCA | 135 |
|  |  | Reverse | CCCTGTTTACTCCCCTGCAG |  |
| Cyclin-like protein | Q6ZIX9_ORYSJ | Forward | AGCGTGGTTGCTGATGAGAA | 123 |
|  |  | Reverse | GGGAGAGATAGCTTGTGCCC |  |
| Elongation factor 1-alpha | B9SPV9_RICCO | Forward | TTGAGATGCACCACGAAGCT | 106 |
|  |  | Reverse | GAAGCAACGAAACCACGCTT |  |
| Fasciclin-like arabinogalactan protein 17 | A9XTM2_GOSHI | Forward | CCGTTGAGTTCTCCACCGAA | 127 |
|  |  | Reverse | CACCTCCGACGAATCTACCG |  |
| Late embryogenesis abundant protein 1 | LEA1_CICAR | Forward | ATTCAGGCCAACCTCAGCTC | 126 |
|  |  | Reverse | TCACTGTGTCCACTGCATCC |  |
| LIM domain protein | Q306K1_BRANA | Forward | GAAAGCAGAAAAGCCCACCG | 129 |
|  |  | Reverse | GTCCCGTTCACCGTAACCTT |  |
| MADS-box transcription factor 1 | D1MFS6_HEVBR | Forward | GCCAGAACTCGTCCCAGATT | 139 |
|  |  | Reverse | TCGCACCCACCAATGTTACA |  |
| Mitotic spindle assembly checkpoint protein MAD2 | D2V3A0_NAEGR | Forward | TCTGAATGGCTGGAAGCTGG | 119 |
|  |  | Reverse | TCGACCACCTCTCCATCAGT |  |
| Nucleotide sugar epimerase | B1WNM2_CYAA5 | Forward | GACCAACCGGCGAGTCTTTA | 127 |
|  |  | Reverse | TTCCCCATGGACCGTAAACG |  |
| Pectate lyase | B6TSP4_MAIZE | Forward | CCCGAAACCTGGGACTCTTC | 118 |
|  |  | Reverse | GGTCTTGTCGCTGGTCATGA |  |
| Polygalacturonase | E3VSV7_CUCPE | Forward | AGCCCAAACACCGATGGAAT | 145 |
|  |  | Reverse | GTCCTGGTCCGCAAGTTACA |  |
| Rho GDP-dissociation inhibitor family protein | D7L724_ARALL | Forward | GCCGTTGAAATCGGTTCGAG | 101 |
|  |  | Reverse | TTGGGCTCTGAAACTTGGGG |  |
| S-adenosylmethionine synthase | B9S0G1_RICCO | Forward | ATTGCCCAAGGAGTCCATGG | 101 |
|  |  | Reverse | TCTGGGGTTTCATCTGTGGC |  |
| Subtilisin-like protease | Q6WNU4_SOYBN | Forward | TCGTCTTGCTGCCTACAAGG | 141 |
|  |  | Reverse | AAGTCTTGAGCTGCGGTACC |  |
| Zinc finger family protein | D7MD22_ARALL | Forward | CACCTCCTTCTTCAGCAGCA | 112 |
|  |  | Reverse | GTCTGTAGGAGAGTGGGGGT |  |

Table S2. Comparing gene expressions between RNA-seq and qRT-PCR platforms

| Gene_Symbol | P_value | Mf_bud_FC | Mf_flower_FC | Ms_bud_FC | Ms_flower_FC |
| --- | --- | --- | --- | --- | --- |
| Actin depolymerizing factor 10 | 0.373356 | -0.03526 | -0.08831 | -0.18702 | 0 |
| Arabinogalactan peptide 20 | 0.227942 | -0.11911 | -2.21314 | 0.492368 | -0.24247 |
| Blue copper protein (Fragment) | 0.089904 | 0.296384 | 2.562836 | -0.18699 | 0 |
| Calcium-dependent protein kinase 2 | 0.123059 | 0.076113 | 0.23137 | 0.50116 | 1.382012 |
| Callose synthase 5 | 0.507366 | -0.20245 | 0.066611 | 0.307915 | -0.8822 |
| CBL-interacting protein kinase 16 | 0.460202 | -0.06802 | -3.75355 | 3.27E-06 | 1.569629 |
| Cyclin-like protein | 0.903404 | -0.15172 | -0.85681 | 0.644802 | 0.549353 |
| Elongation factor 1-alpha | 0.399519 | -0.01383 | 0.740366 | -0.03966 | -0.17512 |
| Late embryogenesis abundant protein 1 | 0.944674 | 0.146182 | 0.830828 | -6.86E-05 | -0.89299 |
| LIM domain protein | 0.044682 | 0.721016 | 5.0188 | 0.561167 | -0.61273 |
| MADS-box transcription factor 1 | 0.589223 | -0.54957 | -1.22755 | 0.026112 | 1.041087 |
| Mitotic spindle assembly checkpoint protein MAD2 | 0.126591 | -0.64093 | 0.329807 | -1.40572 | -0.42123 |
| Nucleotide sugar epimerase | 0.242906 | -0.47722 | -6.10575 | -0.86419 | 1.478928 |
| Pectate lyase | 0.378746 | 0.224552 | 1.404242 | 0.259555 | -0.25448 |
| Polygalacturonase | 0.185593 | 0.454007 | 1.917943 | -0.18715 | -0.39784 |
| Rho GDP-dissociation inhibitor family protein | 0.605447 | -0.0802 | -0.24439 | 0.171454 | -0.22969 |
| S-adenosylmethionine synthase | 0.077719 | 0.321792 | 3.462911 | -0.20851 | -0.0248 |
| Subtilisin-like protease | 0.085363 | 0.793657 | 3.926746 | 0.717842 | -0.89298 |
| Zinc finger family protein | 0.087603 | -0.69539 | -6.00296 | -0.60327 | 0 |
